# Pre-clinical evaluation of small molecule LOXL2 inhibitors in breast cancer

**DOI:** 10.18632/oncotarget.15257

**Published:** 2017-02-10

**Authors:** Joan Chang, Morghan C. Lucas, Lidia E. Leonte, Marc Garcia-Montolio, Lukram Babloo Singh, Alison D. Findlay, Mandar Deodhar, Jonathan S. Foot, Wolfgang Jarolimek, Paul Timpson, Janine T. Erler, Thomas R. Cox

**Affiliations:** ^1^ Biotech Research and Innovation Centre (BRIC), University of Copenhagen (UCPH), Copenhagen, Denmark; ^2^ The Garvan Institute of Medical Research and The Kinghorn Cancer Centre, Cancer Division, St Vincent's Clinical School, Faculty of Medicine, University of New South Wales, Sydney, Australia; ^3^ Pharmaxis Pharmaceutical Ltd., Frenchs Forest, Australia

**Keywords:** lysyl oxidase-like 2, lysyl oxidase, breast cancer, metastasis

## Abstract

Lysyl Oxidase-like 2 (LOXL2), a member of the lysyl oxidase family of amine oxidases is known to be important in normal tissue development and homeostasis, as well as the onset and progression of solid tumors. Here we tested the anti-tumor properties of two generations of novel small molecule LOXL2 inhibitor in the MDA-MB-231 human model of breast cancer. We confirmed a functional role for LOXL2 activity in the progression of primary breast cancer. Inhibition of LOXL2 activity inhibited the growth of primary tumors and reduced primary tumor angiogenesis. Dual inhibition of LOXL2 and LOX showed a greater effect and also led to a lower overall metastatic burden in the lung and liver. Our data provides the first evidence to support a role for LOXL2 specific small molecule inhibitors as a potential therapy in breast cancer.

## INTRODUCTION

Solid tumors remain one of the biggest burdens of disease in the developed world. At present, surgery remains the gold standard for treating patients with resectable disease. Furthermore, early detection is seen as one of the most effective approaches for improving patient survival. Thus, the ability to treat and contain primary tumors carries with it the ability to enhance surgical outcome. There is mounting evidence that changes in the extracellular matrix (ECM) are critical in promoting the growth and metastatic dissemination of solid tumors [1]. Furthermore, many of the biochemical and biomechanical changes in the tumor ECM are a direct result of tumor action [2]. Of late, particular focus has been on the role of matrix remodeling enzymes, and their function in promoting tumor progression [3]. As such, enzymes that post-translationally modify the ECM, and in particular those that cross-link the ECM, such as the lysyl oxidase family are of particular interest as targetable molecules [4].

The lysyl oxidase family of secreted enzymes has garnered much attention over recent years. Consisting of Lysyl oxidase (LOX) and 4 LOX-like enzymes (LOXL1 to 4), this is a family of secreted, copper dependent amine oxidases, which predominantly catalyze the cross-linking of ECM proteins, including collagens and elastin. All of the family members possess a conserved catalytic domain yet exhibit divergent N-termini, which is thought to confer context specific functions. Despite the overlap in sequence homology, there appears to be little reported redundancy in biological function between family members. Yet, several recent papers in the field have implicated many of these family members as potential therapeutic targets, none-less-so than LOXL2. LOXL2 is an 87kDa secreted protein that has been implicated in the progression of solid tumors including breast, pancreatic, biliary, esophageal, prostate, head and neck, colon and gastric cancers [5–12].

Our objective was to evaluate the efficacy of two generations of novel LOXL2 inhibitor in both *in vitro* and *in vivo* models of human breast cancer in order to determine the functional role of LOXL2 in breast cancer progression.

## RESULTS

### The PXS compounds as a new generation of LOXL2 inhibitors

β-aminopropionitrile (BAPN) has long served as the archetypal lysyl oxidase inhibitor, however variable potency, and therefore selectivity, has often been reported. Indeed BAPN, whilst originally proposed to specifically inhibit LOX activity, has also been shown to inhibit LOXL2 activity with a similar potency. This promiscuity, coupled with the lack of amenable sites for chemical modification, has rendered BAPN of little use for clinical drug development and detailed investigations into dissecting the differences in the functional role of LOX and/or LOXL2 in diseases such as cancer and fibrosis.

Derivation of an alternative (haloallylamine-based) inhibitor scaffold has now led to the development of new compounds [13, 14] with different selectivity profiles to facilitate such investigations. These compounds are mechanism-based inhibitors with drug-like properties.

**Figure 1:**
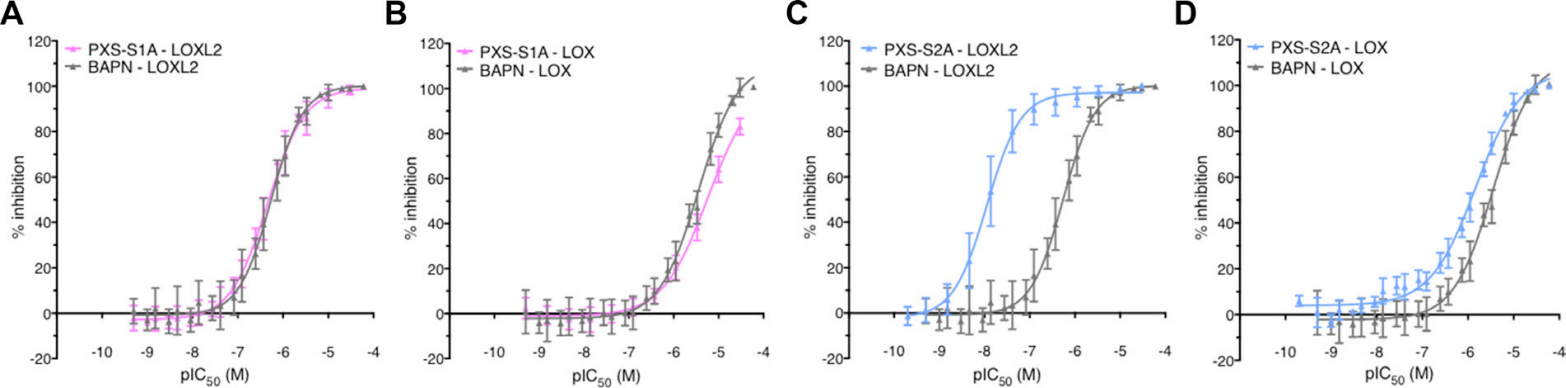
The PXS compounds as a new generation of LOXL2 inhibitors (**A**) pIC50 plots for PXS-S1A and BAPN against recombinant LOXL2 protein and (**B**) native human LOX enzyme showing a very similar activity and selectivity profile (**C**) pIC50 plots for the modified second generation PXS-S2A and BAPN against recombinant LOXL2 protein and (**D**) native human LOX enzyme showing significantly enhanced LOXL2 selectivity.

PXS-S1A is a first generation inhibitor that displays almost identical activity and selectivity (relative to BAPN) when tested against recombinant LOXL2 enzyme (pIC_50_ ± SD, n: PXS-S1A 6.8 ± 0.2; 111; BAPN 6.4 ± 0.1; 45) (Figure 1A) and comparable activity and selectivity (relative to BAPN) to native human LOX enzyme (Figure 1B) (PXS-S1A 5.3 ± 0.3, 3; BAPN pIC_50_ ± SD, n: 5.5 ± 0.1, 8). Given the similarity in affinities for LOX and LOXL2, PXS-S1A represents an incredibly versatile and powerful dual inhibitor of both enzymes. The crucial difference between PXS-S1A and BAPN, however, is the chemical tractability of the former species, meaning that structural modifications can easily be made to improve LOXL2 potency and/or decrease LOX potency, thereby leading to significant increases in selectivity. Such modifications led to the discovery and generation of PXS-S2A, a potent and highly selective LOXL2 inhibitor (pIC_50_ ± SD, n: 8.3 ± 0.1, 49) (Figure 1C) with comparable LOX activity to PXS-S1A (pIC_50_ ± SD, n: 5.9 ± 0.1, 13) (Figure 1D). PXS-S2A does not show any auxiliary pharmacology in standard profiling assays (SafetyScreen 87; Eurofins Panlabs Inc.). It shows excellent *in vitro* properties (high plasma stability and low plasma protein binding) as well as high metabolic stability. The orally bioavailable form of PXS-S2A; PXS-S2B; is readily absorbed following oral gavage, distributes well into tissues and forms PXS-S2A. Safety testing showed that PXS-S2B dosed daily at 10mg/kg over 24 weeks in healthy mice led to no detectable clinical signs.

In terms of selectivity over other related human amine oxidases (semicarbazide-sensitive amine oxidase (SSAO), diamine oxidase (DAO), monoamine oxidase (MAO-A) and (MAO-B)), PXS-S1A exhibited greater than 50 fold and PXS-S2A greater than 500 fold higher selectivity (Table 1). Whilst mechanism-based amine oxidase inhibitors have been reported to potentially serve as substrates for some amine oxidases [15], there was no significant (> 20%) increase of AMPLEX Red signal over baseline for high concentrations (> 30 μM) of PXS-S1A for LOXL2 or MAO-B, although > 20% increases occurred for SSAO and DAO. In contrast to this, PXS-S2A did not show any significant activity against any enzyme tested even at high concentrations (LOXL2, DAO, MAO-B, SSAO).

**Table 1:** pIC_50_ (M) selectivity for PXS-S1A and PXS-S2A against LOXL2, and other related human amine oxidases (semicarbazide-sensitive amine oxidase (SSAO), diamine oxidase (DAO), and monoamine oxidases (MAO-A) and (MAO-B))

	Lysyl Oxidase-like 2(LOXL2)	Diamine oxidase(DAO)	Monoamine oxidase A(MAO-A)	Monoamine oxidase B(MAO-B)	Semicarbazide-sensitive amine oxidase (SSAO)
PXS-S1A	6.8	< 5	< 5	5.1	< 4
PXS-S2A	8.3	< 5	< 5	< 5	< 5

In light of the numerous reports with regard to the critical and differential roles of LOX and LOXL2 in solid tumor progression, in particular breast cancer [3, 4], the availability of a dual LOX/LOXL2 inhibitor (PXS-S1A) and highly specific LOXL2 inhibitor (PXS-S2A), lays the foundation for dissecting the specific functional role of LOXL2 in breast cancer. It also allows the evaluation of the potential of small molecule targeting of LOXL2 as a therapeutic approach in breast cancer.

### PXS compounds inhibit proliferation, migration and invasion *in vitro*

**Figure 2:**
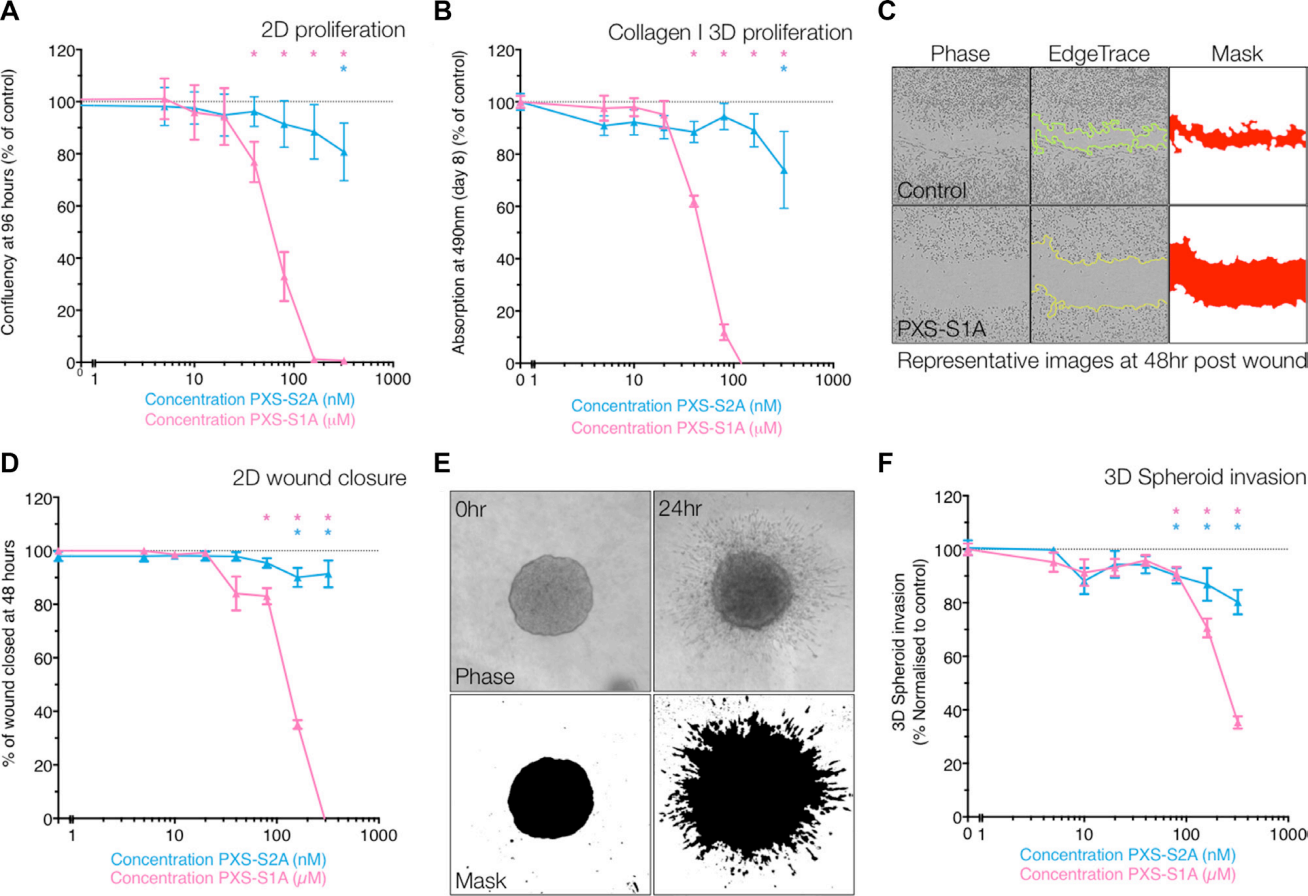
PXS compounds inhibit proliferation, migration and invasion *in vitro* (**A**) Confluency (as % of control) of MDA-MB-231 cells at 96 hours post seeding, in the presence of escalating doses of LOXL2 inhibitors PXS-S1A and PXS-S2A, showing dose dependent inhibition of proliferation. (*n* = 9 measurements over 3 independent experiments). (**B**) Absorption at 490nm (MTS Assay) of MDA-MB-231 cells in 1.8mg/mL 3D collagen I matrices at day 8 (measured as % of control), showing dose dependent inhibition of proliferation in response to LOXL2 inhibition (*n* = 9 measurements over 3 independent experiments) (**C**) Workflow and representative images for 2D scratch wound closure scoring. Using in house ImageJ scripts, first the wound edge is identified, before a mask is applied and the scratch area calculated. (**D**) LOXL2 inhibition leads to a dose-dependent decrease in scratch wound closure (plotted as % of control wound closure) (*n* = 12 wounds over 3 independent experiments) (**E**) Workflow and representative images for 3D spheroid invasion scoring. Using in house ImageJ scripts, a mask is generated of cells and the invaded areas calculated. (**F**) LOXL2 inhibition leads to a dose-dependent decrease in 3D spheroid invasion (plotted as % of control spheroid invasion) (*n* = 12 spheroids over 3 independent experiments).

The efficacy of the LOXL2 inhibitors was tested *in vitro* in proliferation, migration and invasion assays in the MDA-MB-231 triple negative human breast cancer model. These cells express high levels of LOXL2 and moderate levels of LOX, with little or no expression of other LOXL family members (LOXL1, 3 or 4) (Supplementary Figure 1A) [7, 16]. In 2-dimensional proliferation assays on plastic (measured as in change confluence over time with increasing drug concentration), both inhibitors showed dose dependent inhibition of breast cancer cell proliferation over 96 hours, with PXS-S1A exhibiting a greater effect against 2D proliferation than PXS-S2A (Figure 2A). This inhibition of proliferation was also observed in 3-dimensional proliferation assays in 3D collagen I matrices (as measured by MTS assay). Both the dual inhibitor PXS-S1A and the LOXL2 specific PXS-S2A inhibited cellular proliferation in dose dependent manners measured over 8 days (Figure 2B). Our data show that both LOX and LOXL2 play a significant role in 2D and 3D proliferation of breast cancer cells.

In 2-dimensional wound closure (migration) assays on plastic (Figure 2C), the dual inhibitor PXS-S1A significantly affected 2D wound closure in a dose dependent manner, whereas PXS-S2A only showed a reduced, albeit still significant effect, at higher concentrations, suggesting that again, both LOX and LOXL2 are important for 2D migration (Figure 2D and Supplementary Figure 1B). Importantly, we observe that this effect is not as a result of induction of apoptosis (Supplementary Figure 1C) as no differences in apoptosis were observed between inhibitor treated and vehicle control. In 3-dimentional spheroid based invasion assays, both the dual inhibitor PXS-S1A and the LOXL2 selective PXS-S2A inhibited 3D invasion into the surrounding collagen I matrix (Figure 2E and 2F).

**Figure 3:**
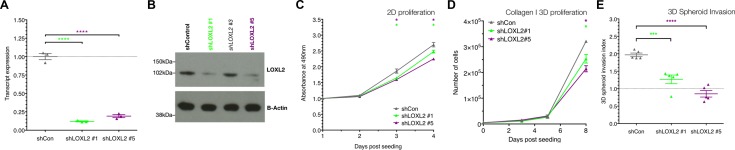
Stable LOXL2 knockdown decreases proliferation, migration and invasion *in vitro* (**A**) qRT-PCR confirms transcriptional knockdown of the *LOXL2* gene with 2 independent hairpins (shLOXL2 #1 and shLOXL2 #5) compared to control (shCon) (*n* = 3 independent experimental repeats). (**B**) Western blotting for LOXL2 protein confirms a reduction in expression levels in hairpins shLOXL2 #1 and shLOXL2 #5 compared to control (shCon). (**C**) shLOXL2 cells show reduced proliferation (MTS assay) in 2D on plastic compared to control shCon (*n* = 9 measurements over 3 independent experiments). (**D**) In 3D collagen I matrices, shLOXL2 cells show reduced proliferation (measured by cell count) over time (*n* = 9 measurements over 3 independent experiments). (**E**) 3D spheroid invasion assays; shLOXL2 cells show a significantly reduced invasive index (normalized to initial spheroid size [dotted line]) compared to control (shCon) (*n* = 9 spheroids from 3 independent experiments).

To confirm the effects of small molecule mediated LOXL2 inhibition in breast cancer cells, we stably knocked down LOXL2 in the MDA-MB-231 cells using separate shRNA constructs. This led to a significant and stable reduction in the levels of LOXL2 mRNA and secreted protein in 2 hairpins (shLOXL2 #1 and shLOXL2 #5) (Figure 3A and 3B). Thus, both of these hairpins (#1 and #5) were used for subsequent validation studies. We also confirmed that stable LOXL2 knockdown did not alter the expression patterns (where detectable) of the other family members (LOX, LOXL1, LOXL3 and LOXL4) (Supplementary Figure 2A). In 2D growth assays on plastic, shLOXL2 cells showed a small and significant difference in proliferation rates (Figure 3C) at day 3 and 4 in line with data shown in Figure 2. In addition to this, in both 3D growth assays and 3D invasion assays in collagen I matrices, shLOXL2 cells showed lower proliferation rates and invasive capabilities when compared to scrambled control cells (Figure 3D and 3E) confirming the effects seen with the LOXL2 specific PXS-S2A on proliferation and invasion of breast cancer cells. Thus, our data show that genetic silencing of LOXL2 mimics the effects of the LOXL2 specific inhibitor, PXS-S2A *in vitro*.

### Targeting LOXL2 *in vivo* delays tumor growth and reduces tumor burden

**Figure 4:**
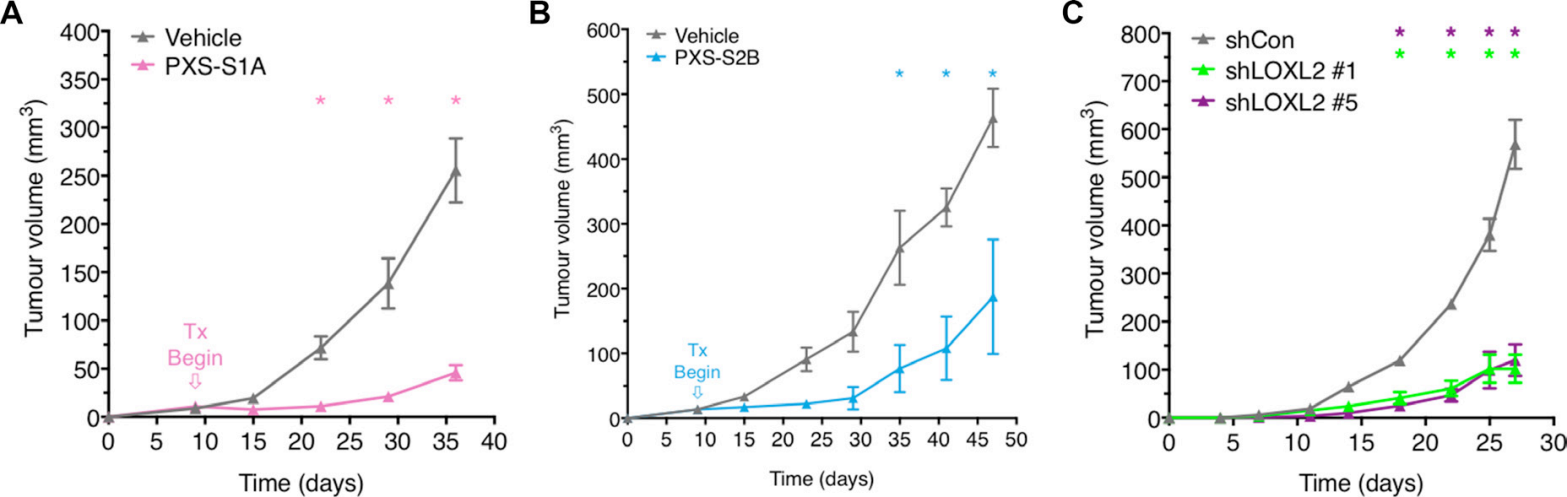
Targeting LOXL2 *in vivo* delays tumor growth and reduces tumor burden (**A**) Treatment of orthotopic MDA-MB-231 human breast cancers in mice with PXS-S1A and (**B**) PXS-S2B significantly delays tumor growth and reduces tumor burden. (*n* = 2 independent experiments; 3 mice) (**C**) Stable silencing of LOXL2 in MDA-MB-231 human breast cancer cells leads to delayed tumor growth and reduced tumor burden in orthotopic models (*n* = 2 independent experiments; 3 mice).

We next tested the efficacy of both generations of inhibitors *in vivo*. MDA-MB-231 human breast cancer cells were orthotopically implanted into the 4th mammary fatpad of athymic Nude (NCR) mice. Once the tumors became palpable, typically measuring 10 mm^3^, and appearing within 7–10 days of implant, treatment with the PXS LOXL2 small molecule inhibitors (PXS-S1A and PXS-S2B [PXS-S2B being the pro-drug version of PXS-S2A used *in vitro*]) commenced. Both generations of inhibitor (PXS-S1A and PXS-S2B) showed clear inhibitory effects on primary tumor growth one week after treatment commenced which continued showing significance at approximately 2–3 weeks post treatment start (Figure 4A and 4B). At experimental endpoints, PXS-S1A showed a ∼75% decrease in primary tumor volume with PXS-S2B showing an ∼55% decrease in tumor volume (Figure 4A and 4B). Our data indicate that the specific inhibition of LOXL2 alone will delay primary tumor growth, suggesting a LOXL2 specific role in primary tumor growth. Dual LOX / LOXL2 inhibition (PXS-S1A) showed a greater degree of inhibition supporting previous studies on the role of LOX in breast cancer primary tumor growth. Our data support the use of both approaches as a potential therapeutic intervention for breast cancer.

To confirm whether the observed effects were as a result of targeting primary tumor secreted rather than stromal-secreted LOX and LOXL2, we utilized our two shLOXL2 MDA-MB-231 cell lines described above. Scrambled control (shControl) and shLOXL2 lines were implanted orthotopically into the 4th mammary fatpad as described above. Stable knockdown of LOXL2 significantly inhibited the growth of primary tumors in the two separate shRNA constructs (Figure 4C). Together these data further support that the targeting of LOXL2 secreted from primary tumor cells is capable of delaying the growth of primary breast tumors *in vivo*.

### LOXL2 inhibition decreases tumor angiogenesis to restrict growth

**Figure 5:**
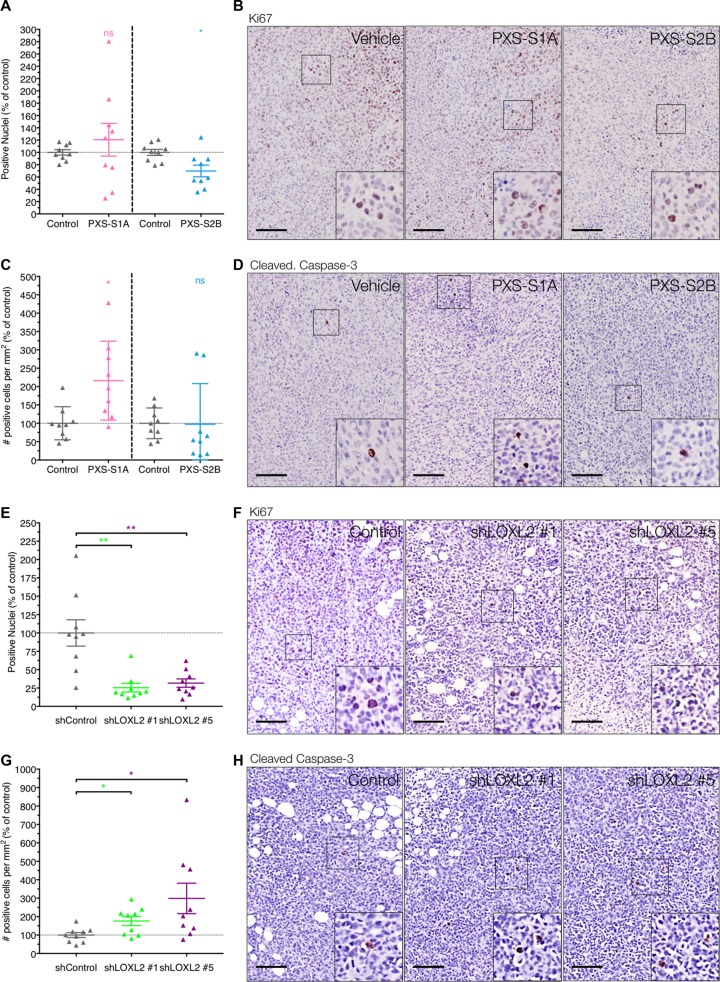
LOXL2 inhibition effects on *in vivo* proliferation and apoptosis (**A**) Immunohistochemistry for Ki67 shows limited effect of the PXS-S1A LOXL2 inhibitor, and a small but significant decrease in Ki67 score by treatment with the PXS-S2B LOXL2 inhibitor *in vivo* (*n* = 2 independent experiments) (**B**) Representative images of Ki67 immunohistochemistry in tumors from control and PXS inhibitor treated mice. (**C**) Conversely, LOXL2 inhibition does not directly induce apoptosis in cells (PXS-S2A), whereas dual inhibition of LOX and LOXL2 (PXS-S1A) shows a significant increase in apoptosis. (*n* = 2 independent experiments). (**D**) Representative images of cleaved caspase-3 immunohistochemistry in tumors from control and PXS inhibitor treated mice. (**E**) Confirmation of findings in shLOXL2 tumors for Ki67 expression shows a significant decrease in tumors Ki67 score *in vivo* following LOXL2 silencing (*n* = 2 independent experiments) (**F**) Representative images of Ki67 immunohistochemistry in tumors from shControl and shLOXL2 implanted mice. (**G**) Genetic silencing of LOXL2 leads to higher apoptosis in tumors as evaluated by cleaved-caspase-3 staining (*n* = 2 independent experiments). (**H**) Representative images of cleaved caspase-3 immunohistochemistry in tumors from shControl and shLOXL2 implanted mice. Scale = 150 μm.

To determine how LOXL2 inhibition may be mediating the significant reduction in tumor volume, sections of orthotopic tumors were assessed for the marker of proliferation Ki67, and the apoptotic marker cleaved caspase-3. Despite being smaller, tumors treated with the PXS-S1A dual inhibitor showed no statistically significant alteration in Ki67 staining (Figure 5A and 5B). However PXS-S1A tumors did show a small but significant increase in the number of positive cleaved caspase-3 cells (Figure 5C and 5D). This is in contrast to the PXS-S2B LOXL2 specific inhibitor, which showed a small but statistically significant decrease in Ki67 staining with no significant changes in cleaved caspase-3 (Figure 5A–5D). This suggests that both generations of inhibitor are altering the balance between proliferation and apoptosis resulting in a reduced tumor burden. To confirm this, we also scored Ki67 and cleaved caspase-3 expression in our stable LOXL-2 knockdown lines when implanted orthotopically. Here we see a more significant effect whereby genetic silencing of LOXL2 in primary tumor cells leads to both a significant decrease in Ki67 expression (Figure 5E and 5F) and an increase in cleaved caspase-3 (Figure 5G and 5H). Given that we saw no change in the levels of other LOX family members upon stable shLOXL2 knockdown (Supplementary Figure 2A), we propose that in this instance, non-enzymatic functions of LOXL2 are likely contributing to the larger differences in proliferation and apoptosis compared to inhibitor-treated mice. However, we hypothesize that the effects of LOXL-2 inhibition, either genetically or pharmacologically, and enzymatic or non-enzymatic, are not direct, but as a result of other changes to tumor physiology given the observed significant reduction in primary tumor volume in both models.

**Figure 6:**
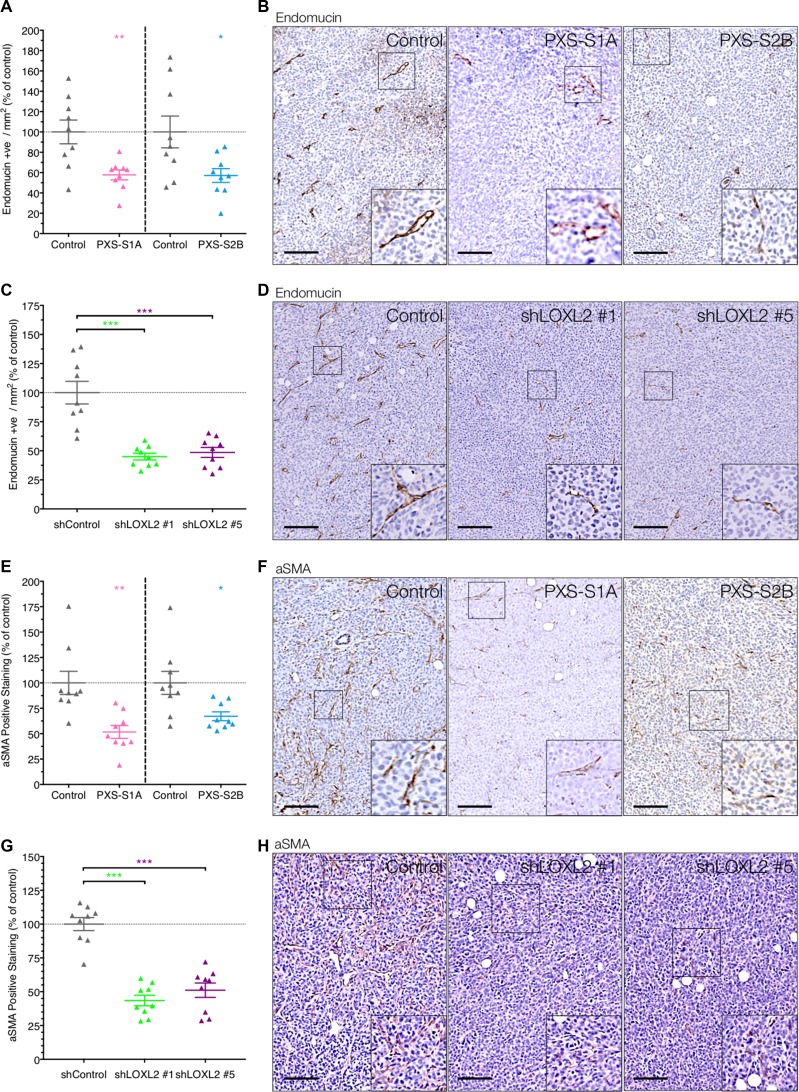
LOXL2 inhibition decreases tumor angiogenesis and CAF activation (**A**) Inhibition of LOXL2 with PXS-S1A and PXS-S2B leads to a significant decrease in vessel density in primary breast tumors *in vivo* (measured by endomucin positive cells). (*n* = 2 independent experiments) (**B**) Representative images of endomucin immunohistochemistry in tumors from control and PXS inhibitor treated mice (**C**) The LOXL2 dependent reduction in vessel density was confirmed in our stable shLOXL2 lines which both show a significant decrease in endomucin positive vessels. (**D**) Representative images of endomucin immunohistochemistry in tumors from shControl and shLOXL2 implanted mice (**E**) Inhibition of LOXL2 using both the PXS-S1A and PXS-S2B compounds leads to a significant decrease in the number of activated αSMA-positive cells in primary tumors (**F**) Representative images of αSMA immunohistochemistry in tumors from control and PXS inhibitor treated mice. (**G**) Silencing of LOXL2 in MDA-MB-231 tumor cells leads to a significant decrease in the number of activated αSMA-positive cells within the primary tumors (**H**) Representative images of αSMA immunohistochemistry in tumors from shControl and shLOXL2 tumor bearing mice. Scale = 150 μm.

One of the biggest limiting factors of primary tumor growth is the availability of nutrients and oxygen as well as the removal of waste products. In order for tumors to expand in size to more than a few mm^3^ they depend on the ability to stimulate angiogenesis, known as the ‘angiogenic switch’ [17]. To that end, tumor angiogenesis has been classed as one of the major hallmarks of cancer [18, 19]. We tested whether the observed significant inhibition of primary tumor growth may be as a result of decreased tumor angiogenesis, which would act to restrict the rate of growth of the primary tumor. Immunohistochemical staining for the endothelial marker endomucin revealed a significant decrease in tumor angiogenesis and vessel count in both the dual LOX/LOXL2 inhibitor treated mice (PXS-S1A) and the LOXL2 specific inhibitor treated mice (PXS-S2B) (Figure 6A and 6B). Importantly, whereas in previous studies we noted a greater effect of dual inhibition, this was not the case for angiogenesis, and so we can conclude that the reduction in vessel density within the inhibitor treated tumors is a result of LOXL2 specific inhibition. Therefore our data show that early inhibition of LOXL2-mediated angiogenesis in developing primary breast cancer leads to the decrease in the growth of the primary tumor.

To further confirm that it was the specific inhibition of tumor-secreted LOXL2 responsible for driving tumor angiogenesis, and not stromal inhibition as a result of systemic treatment with the inhibitors, we also evaluated endomucin scoring in our two stable shLOX2 models. In these models we also see a similar significant reduction in intratumor vessel density, confirming that tumor-secreted LOXL2 promotes primary breast tumor angiogenesis and enhances primary tumor growth (Figure 6C and 6D).

### LOXL2 inhibition decreases cancer-associated-fibroblast activation

We have previously shown that high tumor LOXL2 levels are correlated with an increase in cancer associated fibroblasts (CAFs) within tumors [20]. The activation of stromal fibroblasts to CAFs has been shown to be play a critical role in tumor progression and metastasis, and there are several efforts underway to target CAFs in solid tumors [21]. We hypothesized that in addition to inhibiting angiogenesis, LOXL2 inhibition may also decrease CAF activation. Using αSMA expression as a marker of activated fibroblasts, we confirmed that inhibition of LOXL2 using both the PXS-S1A dual inhibitor and PXS-S2B LOXL2 specific inhibitor led to a significant decrease in the number of activated αSMA-positive fibroblasts *in vivo* (Figure 6E and 6F). Given the emerging importance of the tumor-promoting ability of αSMA–positive CAFs, which has been shown in breast, prostate, pancreatic, and skin cancer mouse models [22–24], the use of LOXL2 inhibitors to target both tumor angiogenesis and CAF activation offers a powerful approach for targeting developing primary breast tumors. Finally, to confirm this, we also evaluated αSMA positivity in shLOXL2 tumors and note a similar significant decrease in shLOXL2 positivity compared to shControl. This further confirms our hypothesis that tumor-derived LOXL2 upregulates αSMA positivity within the tumors.

### LOXL2 as a driver of metastasis

Finally, in addition to angiogenesis, CAF activation and primary tumor growth, we evaluated the effects of the two PXS inhibitors on metastatic dissemination in our human breast cancer model. To do this, primary tumors were implanted into the mammary fat pad as described above and treated with both the PXS-S1A dual inhibitor and PXS-S2B LOXL2 specific inhibitor once primary tumors became palpable (as described above). At experimental endpoints, lung and liver sections were taken, stained with Haematoxylin and Eosin (H&E), and scored for the presence of metastases.

**Figure 7:**
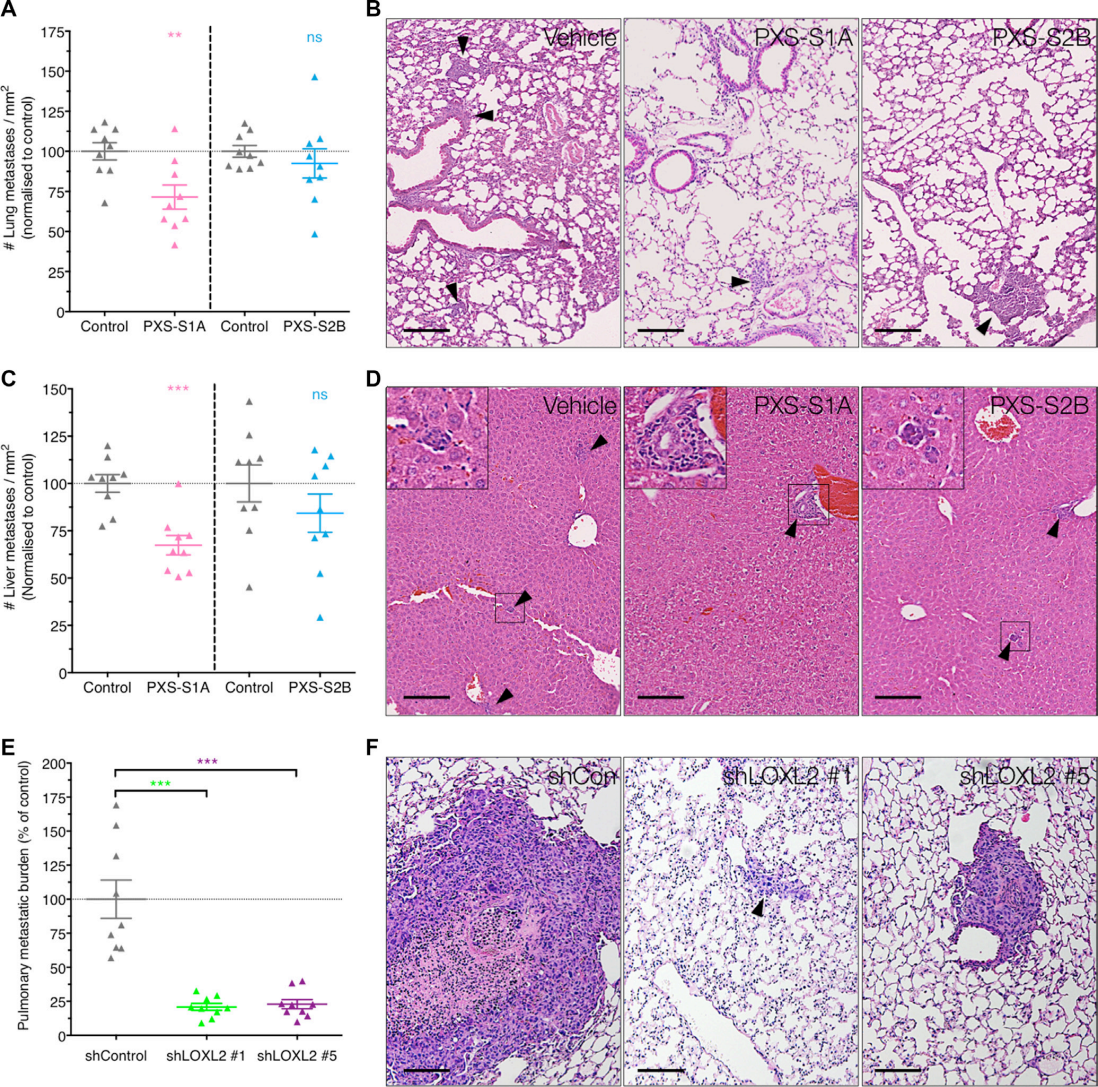
Complete silencing of LOXL2 is required for reduction of metastatic burden (**A**) Treatment with PXS-S1A showed a significant reduction in pulmonary metastatic burden, but not in PXS-S2B inhibitor treated mice (*n* = 2 independent experiments) (**B**) Representative H&E images of pulmonary metastatic burden in control and PXS inhibitor treated mice (**C**) Similarly, inhibition of LOXL2 with PXS-S1A showed a significant reduction in hepatic metastatic burden but this was not significant in the PXS-S2B treated mice (*n* = 2 independent experiments). (**D**) Representative H&E images of pulmonary hepatic burden in control and PXS inhibitor treated mice. (**E**) In contrast, genetic silencing of LOXL2 led to a significant reduction in pulmonary metastatic burden in shLOXL2 tumor bearing mice compared to shCon tumor bearing mice (**F**) Representative H&E images of lungs from shCon and shLOXL2 tumor implanted mice. Scale = 150 μm.

Treatment with the PXS-S1A dual LOX/LOXL2 inhibitor led to a significant reduction in the number of metastases present within the lungs (Figure 7A and 7B) and liver (Figure 7C and 7D). Whilst this trend was repeated in the PXS-S2B treated mice, it was not significant, suggesting that LOXL2 enzymatic inhibition alone may not be fully sufficient to inhibit systemic metastases and that LOX plays a more dominant role in this process, in support of our previously published data [16, 25–27].

However, in contrast, our shLOXL2 stable knockdown cells showed a significant decrease in lung metastatic burden (Figure 7E and 7F). The stable knockdown of LOXL2 will affect all potential LOXL2 mediated interactions, both enzymatic and non-enzymatic protein-protein interactions. Since the PXS inhibitors can only block effects that are due to enzymatic activities, this data indicates that there may also be important non-enzymatic roles for LOXL2 in metastatic dissemination.

## DISCUSSION

Here we present the first data to show that selective targeting of LOXL2 using small molecule inhibitors is effective against primary breast tumor burden. These effects are mediated through decreased angiogenesis and a subsequent reduction in CAF activation.

Previous results have suggested that LOXL2 expression does not affect 2D cellular proliferation [7, 28]; however when embedded in matrix (3D proliferation), LOXL2 overexpression in breast epithelial cells did increase proliferation [28]. In the study presented here, we show that LOXL2 specific inhibition leads to a decrease in 2D proliferation, which also pertains to 3D proliferation. Furthermore, using the dual LOX/LOXL2 inhibitor (PXS-S1A) and the LOXL2 specific inhibitor (PXS-S2A) we show that both LOX and LOXL2 play non-redundant roles in this setting. Our data further show that the effects of LOXL2 activity inhibition on breast cancer cell proliferation and ultimately tumor progression are manifested through an interaction with the surrounding microenvironment, and not necessarily a direct tumor cell response to LOXL2 enzymatic inhibition.

The pro-angiogenic effects of LOXL2 observed in our *in vivo* tumor studies have been further supported by previous research where LOXL2 has been demonstrated to be necessary for collagen IV assembly and organization in the endothelial basal lamina, a process critical to angiogenesis [29]. Indeed, another study using a LOXL2 blocking antibody (AB0023) also showed efficacy against the angiogenic capabilities of endothelial cells, as well as a decrease in microvascular density in tumors [30]. Interestingly, in the latter study the authors showed that despite a reduction in tumor microvasculature, there were no effects on tumor growth; instead, the authors focused on how AB0023 treatment caused vasculature normalization, which in turn allowed for better delivery of therapeutics [30].

The effects on tumor growth observed in our study may also be in part due to the effect of LOXL2 inhibition on reduced angiogenesis as well as CAF-activation, something which was not reported in the 2013 study, determining whether LOXL2 modulation of CAF activation and angiogenesis are mutually exclusive in this context is beyond the scope of this study. However recent reports would imply they are inexplicably intertwined in other solid tumours [31, 32]. Nonetheless, we have previously reported a role for LOXL2 in fibroblast activation through FAK signaling [20], and another study using the AB0023 anti-LOXL2 antibody has also shown that treated tumors exhibit significantly lower levels of activated fibroblasts (CAFs) [33]. These results support our findings here, whereby selective small molecule inhibition of LOXL2 results in a significantly lower numbers of αSMA positive CAFs within developing tumors.

Previous research from both our lab and other labs [7, 33] has identified an important role for LOXL2 in mediating breast cancer progression, and highlighted its therapeutic value. Here, we report the anti-tumor effects of the first selective LOXL2 small molecule inhibitors in models of human breast cancer. Our data strongly support the continued development of selective LOXL2 small molecule inhibitors for use in the treatment of human cancers.

## MATERIALS AND METHODS

### Animals and ethics statement

Female NCR nude mice were purchased from Charles River aged 8 weeks. All experiments were carried out in accordance with and under authorization and guidance from the Danish Inspectorate for Animal Experimentation.

### Cell lines

MDA-MB-231 parental cells (ATCC) as well as shLOXL2 knockdown lines (detailed below) were cultured in standard Dulbecco's Modified Eagle Media (DMEM) supplemented with 10% fetal bovine serum and 0.5% penicillin/streptomycin. All cell lines were routinely tested for mycoplasma and tested negative for murine pathogens by IMPACT testing (IDEXX Laboratories).

### Generation of stable shLOXL2 lines

For stable knock-down of LOXL2 (shLOXL2), HEK293T cells were grown to 40% confluency in T25 cm^2^ flasks and transfected with 1.5 μg of shLOXL2 or shControl lentiviral construct together with 2 μg of packaging vector and 0.67 μg of envelope vector in antibiotic-free DMEM/10% fetal bovine serum, using Lipofectamine 2000 according to manufacturer's instructions. The shLOXL2 and shControl TRC Lentiviral Human constructs are available from Open Biosystems/GE Dharmacon and are targeted against lysyl oxidase homolog 2 [Homo sapiens] NP_002309.1. After overnight incubation, the media was changed to fresh DMEM/10% fetal bovine serum. 24 hours later, the lentivirus-containing media was collected and passed through a 45 μM filter. MDA-MB-231 cells (described above) were cultured to 40% confluency in normal culture media without antibiotics and infected with viral supernatant in the presence of 4 μg/ml polybrene to increase infection efficacy. After 48 hours of infection, polyclonal populations of stably infected cells (MDA shLOXL2#1, shLOXL2#3, shLOXL2#5, shControl) were selected using 2 μg/ml puromycin.

### Proliferation assays

2D proliferation on plastic of MDA-MB-231 breast cancer cells was assessed using both the Incucyte system (Essen BioScience), and CellTiter 96^®^ Aqueous One Solution Cell Proliferation Assay (Promega), according to manufacturer's instructions.

For the Incucyte system, cells were plated in triplicates of 2000 cells per well (96wp) in 100 μl of cell culture media. After overnight incubation, the media was changed to culture media containing either the desired dose of LOXL2 inhibitor, or mock treatment (DMSO); the plate was then incubated and imaged every two hours in the Incucyte system, over a period of 48 hours.

For the 2D CellTiter 96 system, cells were plated at 1000 cells per well (96wp) in 100 μl of cell culture media. After overnight incubation the drugs were added to the plates. For each time-point, 20 μl MTS reagent was added per well and the plate incubated in the dark for 2 hours at 37°C. Absorbance was then measured at 490 nm with an ELISA plate reader (Molecular Devices). All readings were normalized to day 1 readings.

3D proliferation in collagen I of the cells was assessed using either digestion of matrix followed by cell count, or the CellTiter 96 AQueous One Solution Cell Proliferation Assay (Promega), according to manufacturer's instructions. For the CellTiter 96 system, cells were plated in triplicate in 96-well plates at 1000 cells per well in 50 μl of 1.8 mg/ml collagen I (BD Biosciences) mix. For the collagen I mix, rat tail collagen was mixed with 5 × DMEM, ddH_2_O and 23 μl NaOH per ml of collagen I used. The cell suspension was set in a 37°C incubator for an hour before 50 μl of culture medium, containing either LOXL2 inhibitor or with vehicle treatment, was added to each well. The culture medium was changed every 2 days. For each time-point, 20 μl MTS reagent was added per well and the plate incubated in the dark for 2 hours at 37°C. Absorbance was then measured at 490 nm with an ELISA plate reader (Molecular Devices). All readings were normalized to day 1 readings.

For the matrix digestion method, 5000 cells were plated in triplicates in 24-well plates in 1 ml of 1.8 mg/ml collagen I (BD Biosciences) mix. At each time point, 1mg/ml collagenase I was added to each well and incubated at 37°C for an hour, after which the number of cells in the digested suspension were counted using a cell counter.

### Hanging drop assays

The invasive capability of the cells was assessed using the hanging drop invasion assay. Spheres of cells were created by the hanging drop method described as followed: cells were trypsinized and resuspended at 400,000 cells/ml in culture media with 0.24% methylcellulose. The lid of a 10cm dish was inverted and 20 μl drops of the cell suspension were aliquoted on the inside of the lid, after which the lid was carefully placed back on the dish, such that the drops of cell suspension were hanging off the lid due to gravity. After 24 hours of incubation at 37°C, spheres of cells aggregated at the bottom of the hanging drop. In a 48-well plate, 500 μl of 1.8 mg/ml collagen I mix (described above) was added to each well and allowed to set for 5 minutes, before the spheres were carefully picked up using a 10 μl pipette and placed onto the half-set collagen mix. This was to ensure the spheres would not sink to the bottom of the plate and come into contact with the plastic surface. 4 spheres were placed into each well with ample distance between each. The collagen was then allowed to set completely by incubating at 37°C for 1 hour, before the culture media (with LOXL2 inhibitor or vehicle treatment) was added and the spheres imaged immediately (T = 0) using a light microscope (Leica DM1L). The plates were then incubated at 37°C overnight, before another set of images were taken to assess the spread of invasive cells into the matrix.

Using ImageJ64 software a mask of the spread of cells was captured and areas were measured by generating a spheroid mask at all time points. An invasive index was calculated by calculating the ratiometric change in the mask area measured between timepoints.

### 2D scratch wound assay

2D migration and wound healing was carried out using a standard assay. Briefly, cells were plated in 96 well plates and grown to 95% confluency. A single scratch wound was then created in each well using a 96-pin woundmaking tool (WoundMaker^™^ - Essen Bioscience). Following wounding cells were washed twice with pre-warmed media and fresh media applied with either PXS inhibitors or vehicle control. Wounds were then imaged using an IncuCyte (Essen Bioscience). Images were then exported to ImageJ where an in house script was used to first define the wound margins and a mask applied to calculate the wound area.

### Apoptosis assay

Apoptosis assays were carried out using the Cytotox Red apoptosis assay (Essen Biosciences) according to manufacturers instructions.

### Primary orthotopic tumor model

Adult female immunodeficient nude mice (NCR nude) aged between 7–8 weeks old and weighing between 19–25 g were used for orthotopic studies. 1 × 10^7^ cells were resuspended in 100 μl of sterile PBS and injected into 4th mammary fat pad on the right flank of the animal. Once the tumors were palpable (approximately 10 mm^3^ in measurement), drugs re-suspended in 2% Tween80/ddH_2_O (PXS-S1A at 2.5 mg/ml (dosed at 10 mg/kg); PXS-S2B at 1.25 mg/ml (dosed at 5 mg/kg)) were administered daily through intraperitoneal injection. Tumor growth rate was measured every 3 days using calipers, and all mice were sacrificed when one of the tumors reached approximately 1000 mm^3^ in size. The tumors, lungs and livers were excised and fixed in 4% PFA; these were then paraffin-embedded, sectioned and stained with haematoxylin and eosin, or used for immunohistochemistry.

### Immunohistochemistry

PFA-fixed tissues were embedded in paraffin according to standard histopathology approaches. Tissues were then sectioned at 4 microns. Sections were deparaffinized with xylene and rehydrated by immersion into decreasing concentrations of ethanol. Slides were then subjected to antigen retrieval by boiling in antigen retrieval buffer (Dako) for 20 minutes, and cooled to room temperature. Endogenous peroxidase activity was quenched through incubation in 3% hydrogen peroxide in methanol. Sections were washed 3× in PBS and blocked with 5% goat serum/2% BSA/PBS for 1 hour and incubated overnight with primary antibodies diluted in 2% BSA/PBS at 4°C. Primary antibody dilutions were as follows [Endomucin Santa Cruz (sc-65495) IHC – 1:100; Activated caspase-3 Abcam (Ab2302) IHC – 1:100; Ki-67 Leica Microsystems (NCL-Ki67p) IHC – 1:100; αSMA Abcam 1A4 (ab7817) IHC 1:100]. Control slides with no primary antibodies added were incubated with just 2% BSA/PBS. After washing 3× with 0.1% BSA/PBS, slides were incubated with respective anti-mouse or anti-rabbit biotinylated antibodies (Dako) for an hour. Slides were then washed 3× again with 0.1% BSA/PBS before visualization with either 3,3′-diaminobenzidine (DAB)(Dako) or Novared (VWR). Slides were then rinsed in H2O and counterstained with haematoxylin. After mounting and drying, slides were scanned using the Hammamatsu digital slide scanner, and quantification of positive staining was carried out using Image J64 analysis and in house scripts.

### qRT-PCR

Cells were lysed in Qiagen RLT buffer and homogenised with QIAshredder following manufacturer's instructions. RNA was extracted from the lysates using the RNeasy Mini Kit and treated with DNaseI according to manufacturer's instructions. Template cDNA was generated using Omniscript reverse transcriptase according to manufacturer's instructions in the presence of RNaseOUT, and subsequently used for amplification. The PCR was performed using an Applied Biosystems 7900HT Fast Real-Time PCR system (Applied Biosystems, Carlsbad, CA). Gene expression for LOX, LOX1, LOXL2, LOXL3 and LOXL4 and GAPDH as control was measured using commercial TaqMan assays (Applied Biosystems) according to manufacturers specifications.

### Western blotting

Immunoblotting for LOXL2 was as previously described [7, 20, 26, 28]. Immunoblotting for LOX, LOXL1, LOXL3 and LOXL4 was as previously described [26]. Primary LOXL2 antibody (Ab55470) was used at 1:1000 and b-actin (Abcam) at 1:10,000, with primary antibody incubation overnight at 4°C. The primary LOX antibody (synthesized by OpenBiosystems) targets a conserved peptide sequence from the active site of human and mouse LOX proteins and has been shown not to bind other LOX family members [25, 26, 34]. The LOX antibody was used at 1:500 dilution. The LOXL1 antibody (Abcam (AB87748)) was used at 1:100 dilution. The LOXL3 antibody (Abcam (AB171315)) was used at 1:200 dilution. The LOXL4 antibody (Santa Cruz (sc-66952)) was used at 1:100 dilution. Vinculin (Sigma) was used at 1:500 dilution. Species-specific HRP secondary antibodies (Dako) were used at 1:30,000 and incubated for 1 h at room temperature, and visualization performed using ECL Plus (Amersham, GE Healthcare).

### Activity/inhibition assays

The standard amplex red assay has been used as previously described [14] using putrescine as a substrate and native ´fibroblast derived LOX, which was prepared by collecting the supernatant of IMR90 cells, followed by a size filtration > 10 kDa for concentration and < 50 kDa to separate from LOXL2, or recombinant LOXL2 as enzyme (R&D Systems).

### Data analysis

Data was analyzed using the Student t test unless otherwise stated, and considered statistically significant when the P value was less than 0.05. Graphs represent the mean and standard deviation. Details for replicates are given in Figure legends. Statistical significance representations are as follows: *P < 0.05, **P < 0.01, ***P < 0.001. Statistical analyses were performed using Prism statistical package version 6.

## SUPPLEMENTARY MATERIALS FIGURES


