# Supplementary material for: Pre-clinical evaluation of small molecule LOXL2 inhibitors in breast cancer
Source: Oncotarget. 2017 Feb 10;8(16):26066–78. doi: 10.18632/oncotarget.15257 (PMC5432238; doi:10.18632/oncotarget.15257)
Supplement: Supplementary file 1 [file oncotarget-08-26066-s001.pdf]

Pre-clinical evaluation of small molecule LOXL2 inhibitors in breast cancer

Supplementary Materials

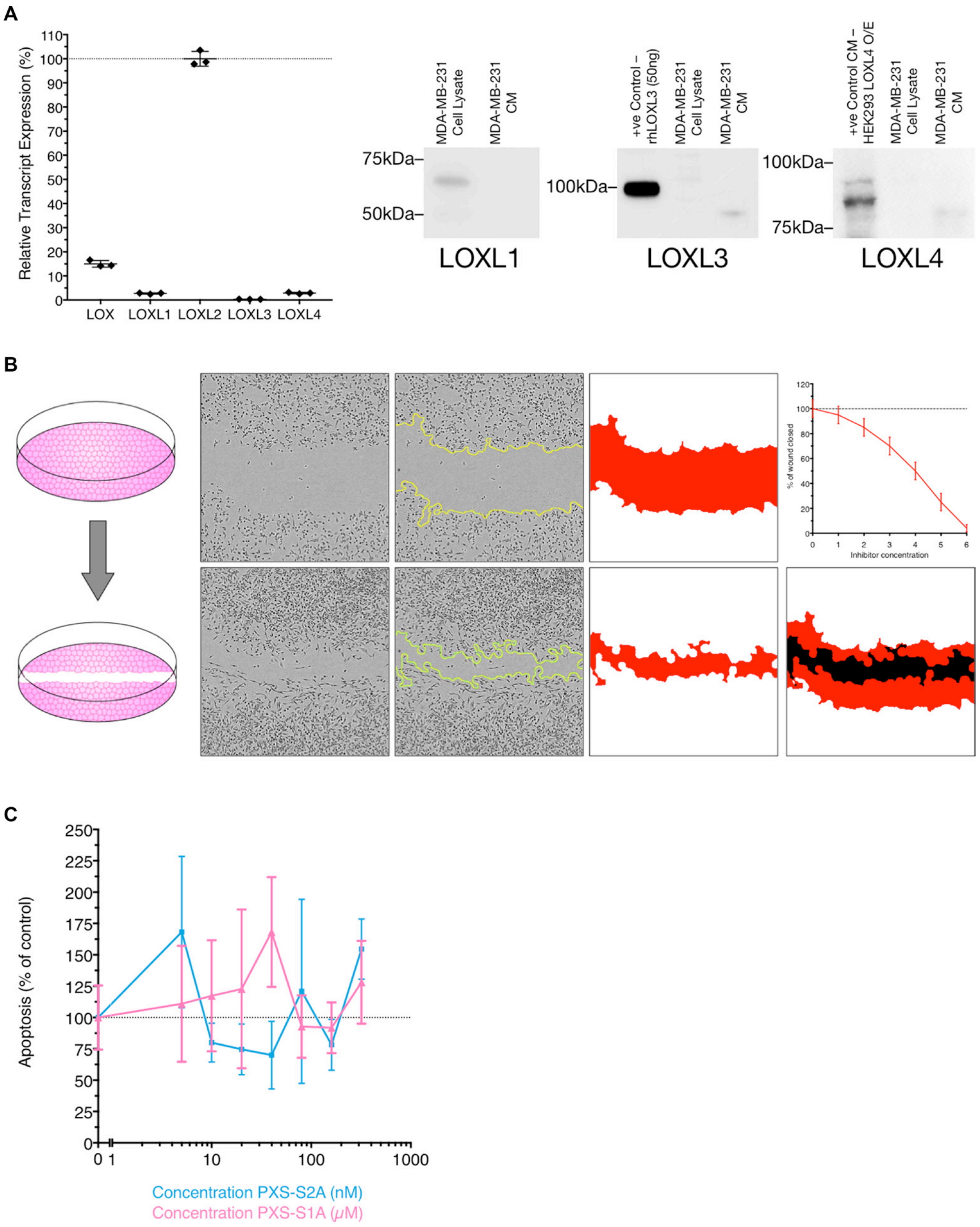

Supplementary Figure 1: (**A**) (left) Q-RT PCR for lysyl oxidase family members (LOX, LOXL1, LOXL2, LOXL3 and LOXL4) in MDA-MB-231 human breast cancer cells. LOXL2 is the most abundantly expressed transcript with LOX being the second most. Levels of LOX1, LOXL3 and LOXL4 are barely detectable at less than 3% of LOXL2 levels. (right) levels of LOX1, LOXL3 and LOXL4 are barely detectable at the protein level. (**B**) Schematic overview of scratch wound assay. Cells are plated and grown to confluency. Once confluent a single scratch is made and imaged. Successive imaging is then carried out at defined timepoints. Wound closure rates are then calculated using in house scripts in ImageJ. To begin, the wound edge is identified. A mask is then applied to calculate the area remaining between wound edges. Timepoint masks are then overlaid and % closure calculated. (**C**) Cytotox Red apoptosis assay shows no difference in levels of apoptosis between the PXS-S1A / PXS-S2A inhibitors and vehicle control. Data have been normalized to vehicle control (100%).

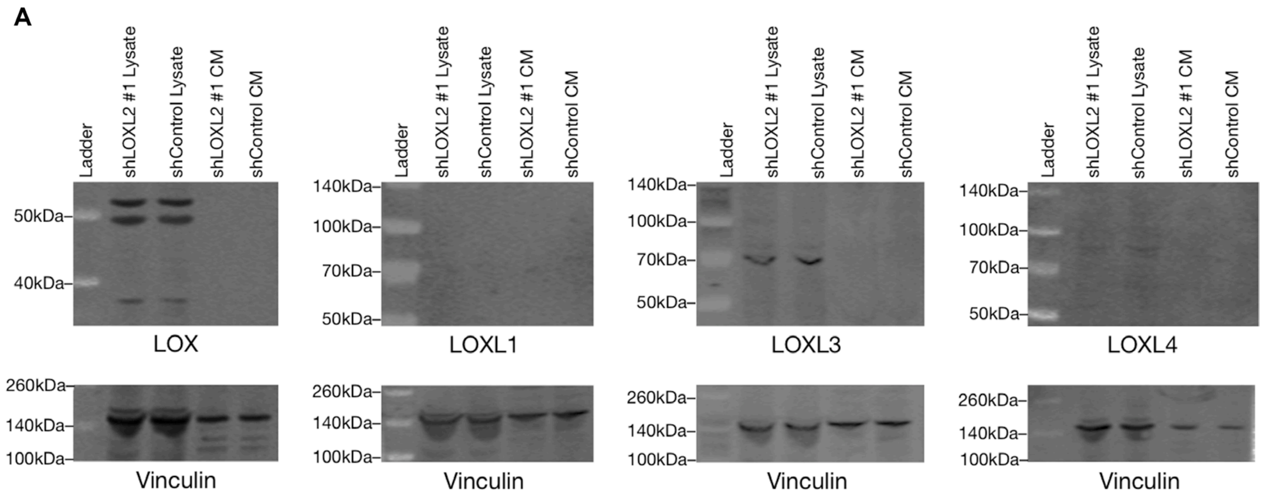

Supplementary Figure 2: (**A**) Stable knockdown of LOXL2 in the MDA-MB-231 cell line leads to no detectable changes in the expression of LOX, LOXL1, LOXL3 or LOXL4 in either cell lysates or conditioned media.
